# The Retention of Devitalized Teeth without Danger of Focal Infection*Read before the Section on Stomatology at the Sixty-Eighth Annual Session of the American Medical Association, New York, June, 1917. Reprinted from *Journal A. M. A.*, September 22, 1917.

**Published:** 1917-10

**Authors:** M. L. Rhein


### THE RETENTION OF DEVITALIZED TEETH WITHOUT DANGER OF FOCAL INFECTION *

M. L. RHEIN, M.D., D.D.S.

The accumulated evidence, both in research work and from clinical data has established the fact that many infective lesions of vital parts of the body can be traced to focal infections at the ends of the roots of teeth. The great amount of attention that has been brought to this important subject has resulted in the usual exaggeration, not only by the laity, but by many physicians. Consequently, it is timely that it should be made very clear that all cases of endocarditis, arthritis, ulcers of the stomach, etc., are not of dental origin.

The value of the natural teeth in the maintenance of health should not be overlooked, and they should not be extracted in a wholesale manner on insufficient evidence of their being a menace to life.

It should be understood that the evidence of a roentgenogram requires corroboration, that poor roentgenograms are apt to be most common, and that a picture in which the tissues can not be histologically studied is unreliable.

The correct reading of such a picture for the purpose of making such a diagnosis is dependent on the definition and detail of root, alveolar process and lining membranes being absolutely clear. Even then it may be necessary to obtain other pictures at varying angles, and finally only a man thoroughly familiar with the histologic appearance of these tissues is capable of a fair interpretation of the same. Even when a roentgenogram has demonstrated the presence of focal infection in the alveolus, it does not prove it to be the etiologic factor sought for. On the

* Read before the Section on Stomatology at the Sixty-Eighth Annual Session of the American Medical Association, New York, June, 1917. Reprinted from *Journal A. M. A.*, September 22, 1917.

other hand, it may be a contributory cause together with some other focal infection.

Too little attention in this period of quick action is being given to the making of a differential diagnosis. In this respect the value of the streptococcus viridans complement fixation test is worthy of serious attention. In view of the fact that more than one hundred and fifty strains of this group have so far been isolated, we must bear in mind that if it has a value, a negative result is by no means conclusive that a later test with some new strain may not show a positive fixation. I am firmly convinced from corroborating clinical evidence that the test has a certain diagnostic value. When a positive result is obtained there remains the self-evident fact of the value of an autogenous vaccine from this source.

Whenever a diagnosis of a focal infection at the end of a root has been made we must give due consideration to the question of immunity. The transitory character of immunity must never be forgotten, just as we must remember how much we still have to learn about immunology. Consequently, it follows naturally from this, that given a focal infection in the alveolar region, it must, as a question of prophylaxis, be cured or the tooth should be extracted.

Where corroborative evidence points to this pathologic site being the cause of metastatic disturbances of a serious nature, it should prompt the cure of the infection as rapidly as possible. In such cases immediate extraction of the diseased tooth is frequently made necessary on account of the time required to eradicate such an infection and leave the tooth in a condition free from the danger of reinfection.

On the contrary, where no metastasis is discovered it leaves the dentist ample time to attempt to properly preserve such a diseased tooth.

Perhaps the greatest reason why this operation is done so imperfectly, even at the present time, by very capable men, is on account of the fact that in the majority of cases it is one of the most difficult of operations and often requires many hours of patient labor divided into different periods of time. This is due to the fact that in so many cases calcification has blocked the root canals, and it makes no difference how extensive this calcification has become, it is essential that every canal must be explored until a broach can pass through the foramen. This can only be accomplished by Roentgen-ray checking at every stage of the operation. It is most difficult to have the average operator understand that certain definite results must be attained regardless of time consumed.

I have been frequently criticised for the length of time I have taken in certain cases, but would state here that perhaps these men, many of them thoroughly conscientious, would think further if they could see some of their failures that had been rectified, failures that were due entirely to their unwillingness to take the time necessary to explore canals to their end. The great expense, necessitated by the amount of time consumed, prohibits the operation in a great many cases. Too many men are easing their conscience by doing better work than formerly, and yet not obtaining correct results. This, however, should not suffice any longer; the principles are now understood, the Roentgen ray reveals the result, and if we make any pretense to honesty we must cease allowing imperfect root canal work to remain in the mouths of people who leave the dentist deluded into thinking that the disease has been eradicated. Remember, after all has been said, that our first duty is not to ourselves, not to our colleagues, but to the community at large.

With this understanding let us consider the principles, technic and final result to be attained.

The first thing to remember is that every step and stage of our work must be done under the most thorough aseptic surroundings. The mouth should be thoroughly sprayed at the outset of every sitting. The gingival border of the root to be operated on should be painted with iodin and the rubber dam should invariably be applied.

It is not feasible here to elaborate the varying details of removing pulp tissue from roots that deviate from the normal type and that exhibit different pathologic characteristics. Let it suffice at this time to say that every particle of organic pulp tissue must be removed, however intricate and tortuous the canals may be; all bay-like rescesses and divergent canals must be cleared of organic matter. Unless this can be accomplished it is useless to proceed further.

When it is desired to make a complement fixation test, the utmost care in the way of asepsis should be taken in regard to the removal of pulp tissue. No chemicals of any kind can be employed on account of the danger of destroying any existing bacteria. When the roentgenogram shows that a sterile broach can be passed through the foramen, a fine iridoplatinum wire previously heated is passed through the foramen and turned around a number of times in the periapical region. This is now withdrawn with care so that the inoculated wire should come in contact with nothing prior to passing into an agar-agar tube, or better still, in a sterile broth tube. The carefulness of our technic alone will determine the value of the culture produced.

The therapeutic agents used to remove pulp tissue are the sodium and potassium alloy and dilute sulphuric acid. No therapeutic agent should be used in the canal which will affect any pathologic condition, which might otherwise interfere with a correct diagnosis. This especially precludes the use of all preservatives such as creosote or the phenols, which will in addition prevent an ideal hermetical sealing of the canals at a later stage.

The galvanic current is now brought into use for the purpose of zinc ionization. Without going into the details of ionic therapeusis, it can safely be said that all of the infected area can be made sterile in this way. It is all dependent on the amount of ionization used. Where there is any uncertainty as to periapical sterility, it should be determined by taking a culture from this area which should show absolute sterility. This necessarily means a delay of at least ten days when, if no culture has shown, the part should be again sterilized prior to sealing the canal. So far no investigation has shown how long such an area will remain sterile. Until this has been definitely solved, it is important that the root canal sealing should immediately follow the last ionization of a given part. In multi-rooted teeth the ionization should be carried on through each separate canal which should immediately be filled before another one has been ionized.

Every step having been so far satisfactorily carried out, we reach the final and most important stage of the operation, that of the root canal filling. This material should be nonirritating, unchangeable in character, and should so homogeneously and hermetically seal the interior of the canal as to safeguard against any infection through the tubuli from the cementum. Its most important function, however, is to seal the outside of the end of the root against two important factors. The first one is to defend the slightest opening of a foramen again the invasion of bacteria. The second, and equally important factor, is to hermetically seal any exposed surface of cementum or dentin against osteoclastic invasion.

The encapsulation of the end of the root with gutta-percha accomplishes both of these purposes. No stage of the operation requires so ideal a maintenance of asepsis.

The proof that such an operation as above described will eradicate the disease, consists in the fact that this granuloma area disappears and is replaced by new alveolar structure.

Anything that interferes with the success of these steps is liable to make the operation a failure.

The most common cause of failure among the men skillful in this operation is an imperfect encapsulation, which oftentimes can not be detected until it is discovered that the new bone has not regenerated.

It does happen that regeneration of bone proceeds for a given time, perhaps for a year, and then on account of imperfect encapsulation, a new infection has started up, showing by evidence of new rarefication; or absorption of the root starts in at some point because there has been an exposed point of cementum not sealed by guttapercha.

On this account it is both unwise and not safe to be positive of success until the part has been under roentgenographic observation for a couple of years. Patients should be instructed to come back for these pictures at least every six months until assured that the new bone is a permanent addition.

If it is found impossible to effect the permanent regeneration of new bone in this way there still remains a final operation by means of which the root may be maintained in a healthy manner.

The amputation of the diseased end of the root and thorough curettement of the periapical region has become a well-recognized procedure, but should be a measure of last resort.

Many apicoectomies brilliantly done eventually turn out failures, because of root absorption due to the action of iconoclasts on the exposed cementum or dentin on the stump of the root.

This should be obviated by permanently insulating this dentin surface from the body tissues. This has been done in the past by an amalgam filling over the stump which entirely covers it. An easier method and one more compatible to human tissue is to dry the surface of the stump and cover it with a few coats of a chloroform solution of guttapercha. The rapid volatilization of the chloroform produces a dehydration of the surface and the guttapercha is left firmly glued to the end of the root.

Of course the same follow-up roentgenograms must be taken after this operation. When none of these methods produce permanent alveolar regeneration it becomes our duty to see that the tooth in question is extracted.

### ABSTRACT OF DISCUSSION

Dr. M. I. Schamberg: It is evident that an immense amount of work is being done to clarify the atmosphere as far as focal infections are concerned. I fear that too little attention has been given to the areas of infection that occur in the mouth that do not show in roentgenograms; in other words, where teeth are pulpless and show no evidence of infection at the end until they are removed and the tips of the roots snipped off and cultured. The same thing applies to the cases of incipient gingivitis which have not really reached the stage of having become definite cases of pyorrhea; cases in which there is no pus discharge, but in which there is, however, a breeding space for the lodgment of organisms that are a menace to the health. The mouth is definitely the slum district of the body, and in that mouth we have all the criminal organizations that tend to invade the system, and produce the havoc that we have heard so much about. In spite of excellent work done by Dr. Rhein and his followers, I am inclined to favor only a more definite method of curing apical infections—that the vast majority of practitioners be not permitted to deal with pulpless teeth. I believe that at least seventy-five per cent. are failures in spite of all that we have learned, and these conditions are a great menace to the health where they are directly or indirectly responsible for the numerous secondary involvements.

Dr. T. W. Brophy: The most essential step to be taken in the prevention of these diseases is the training of parents as to hygienic methods. When the time arrives, Dr. Atkinson used to say, that the members of the profession and the public are brought to realize the serious consequence of dental caries, and its sequelae, then we will have arrived at a time when these diseases will be less numerous. I was further impressed with the feeling that the class of infections that have been discussed constitute a class of infections and diseases that has not received the attention that its importance demands on the part of medical teachers. One of the greatest difficulties with which I have had to contend is to find that interns, who are among the best educated of the medical graduates, have never had instruction, with few exceptions, on the diseases incident to the teeth; when they come in touch with these maladies they are not qualified to make a diagnosis or to express an intelligent opinion as to the best course to pursue in the management of their patients.

Dr. Frederick B. Moorehead: There are certain fundamental facts that we must all face in this whole question. First, the fact of focal infections as an established truth in pathology; second, the incidence of chronic mouth infections, in relation to general infections; third, we must all agree that any infection, wherever found, should be removed. These are postulates which we must accept as a working basis. Now, the question arises, how shall this be done? For the most part by the extraction of teeth.

Dr. Rhein has indicated the possibility in the hands of an expert of saving a few teeth. He failed to tell us how many times he fails to do the thing he showed us on the screen this morning. I know he has a good percentage of failures, and he removes the teeth when treatment fails. An expert giving a lot of time in saving a few teeth does not determine an average for the profession. I am perfectly willing to admit that where teeth can be handled by a man as expert as Dr. Rhein, badly infected teeth may be saved in many cases. A specialist has many advantages over the general practitioner, both in opportunity and the class of patients who come to him. Where the patient's health is not put in jeopardy, while infected teeth are being treated, and where the case is carefully checked up by the Roentgen ray, no one has any complaint to make; one may develop a highly specialized technic in root canal management if he is willing to give thought and attention to it; but how many have developed such skill? Not many! How many people are there who may be served by that type of men? What about the great population of foreigners? What about the poor? What about the people who are bedridden in our hospitals; what about people suffering from general chronic infections? Are we going to subject them to any detailed regime? Certainly not. Reduced to fundamental facts, it becomes evident, after all, that, for the most part, teeth that are badly involved in chronic infections will have to be extracted.

Dr. Rhein: I am in thorough sympathy with what Dr. Moorehead has so well said in a general way, except that I think he goes a little to extremes. I think he overestimates the lack of men who are doing good root canal work at present. I know that personally I have trained and educated over two hundred men in this very district that we reside in, who are doing first-class root canal work. There is a great deal that he has said, however, that it is not thought advisable to debate on. I would especially refer to the difference, the unwillingness of a man, after he has spent two hours, or twenty hours, on a good tooth—and that has happened many times with me—then to tell the patient that the operation is a failure and that the tooth must be removed. That is the difficult point to make the dentist understand. It is a simple thing with the surgeon, and his confrere, the physician. Dentists have been brought up to look at these things as jobs, mechanical jobs; and they are afraid to tell their patients that the operation is a failure and that in the interest of their health the tooth must be removed. That is the point I want to emphasize in regard to what Dr. Moorehead has said.

Dr. Kurt H. Thoma: I am connected with an institution which deals with chronic infectious cases, and most of the patients suffer from chronic arthritis of long standing. Our hospital is in a position to give these patients the very best attention in all respects.

From the beginning I have practiced radical extraction of all abscessed teeth, and in order to restore the patient's masticating efficiency, which is, of course, of great importance, we have a dental intern who supplies plate and bridge work after the extractions.

The dental department of the hospital has existed for about two years and in looking back on the many cases under our observation we find that we did not get the results which we first expected. We thought we could cure a large percentage of the patients and, although we have not done this, we find improvement in many cases. The acute symptoms of pain and swelling have improved, but they have not been entirely corrected. This is due to the fact that the destruction in the joint, or other part, where the infection is located, has progressed to such an extent that anatomic restoration is impossible. However, we do look for improvement of a more physiologic nature. The purpose of correcting abscessed teeth, infected tonsils and intestinal disorders is to improve the patient's general condition and from this and improved nutrition, in which masticating efficiency plays an important part, we expect improvement of the chronic disease. We find that we are able to put the patient in a condition which, though not ideal, enables him to do some kind of work to a limited extent, thus making a self-supporter out of a man who would otherwise be a burden to the community.

